# Arthroscopic reduction of adult comminuted tibial eminence avulsion with single tunnel pull-through suture fixation: A case report of technical note

**DOI:** 10.1016/j.ijscr.2022.107616

**Published:** 2022-09-09

**Authors:** Hudaya Nikmatullah, Kukuh Dwiputra Hernugrahanto

**Affiliations:** a Fellowship of Indonesia Hip and Knee Society, Department of Orthopaedic and Traumatology, Dr. Soetomo General Hospital, Surabaya, Indonesia; b Department of Orthopaedic and Traumatology, Faculty of Medicine, Universitas Airlangga/Dr. Soetomo General Hospital, Surabaya, Indonesia

**Keywords:** Comminuted eminence fracture, Tibial eminence avulsion, Suture fixation

## Abstract

**Introduction and importance:**

Tibial eminence avulsion is an exceptional knee injury in the adult population. The treatment of this injury is generally based on the pattern of the fragment fracture displacement and operative treatment is recommended for comminuted eminence fracture to prevent nonunion and knee instability.

**Case presentation:**

We reported a 30-years-old female with severe left knee pain after falling from a motorcycle. Physical examination showed moderate left knee effusion with restricted knee joint range of motion from 0 to 90°. Anterior drawer test was positive without opening varus or valgus stress. A preliminary radiograph and CT scan of the left knee denoted displaced and comminuted tibial eminence avulsion.

**Clinical discussion:**

Anatomical reduction of the displaced fragment and adequate tension of the ACL bundle are the surgical goal to promote fracture healing, maintain knee stability, and restore range of motion. Various reduction and fixation techniques have been devised for comminuted tibial eminence avulsion ranging from arthroscopic screw to suture fixation. Although arthroscopic reduction and suture fixation become the prominent treatment for this injury, the ideal technique remains unclear.

**Conclusion:**

We propose a modified arthroscopic reduction and suture fixation technique by using a single tibial tunnel and knotless distal anchoring screw fixation. Despite some limitations, this technique simplifies anatomic reduction and fixation with single tunnel placement which has shown to be effective in reducing surgical time.

## Highlights

• Operative treatment is highly recommended for comminuted tibial eminence avulsion fractures.
• Arthroscopic-assisted reduction becomes the treatment of choice in these injuries.
• But the most optimal procedure of arthroscopic reduction and suture fixation technique still remains uncertain.

## 1 Introduction

Tibial eminence fracture is caused by ACL avulsion at its insertion site [1]. The mechanism of this injury is identical to the ACL rupture, except that instead of tearing off the ligament fiber, a piece of the eminence fragment is pulled from the tibia along with the ACL [2]. This injury is commonly encountered in children and adolescents, and its incidences are equivalent to the ACL rupture in adults [3]. However, this condition is not only reported in those populations. Although less common, adults can also suffer from tibial eminence fractures [4] and are most commonly associated with high-energy trauma such as road traffic accidents [5]. In adults, tibial eminence fracture is frequently accompanied by meniscal, capsule, or collateral ligament injuries. Recently, this injury has become more common in the adult population [3].

According to the severity of tibial eminence fragment displacement, Myers and McKeever [6] classified these fractures into 4 types. Type I with no or slight fragment displacement. Type II fracture in which the anterior edge of the eminence fragment is uprooted with the posterior hinge still intact. Type III fracture is a totally displaced fracture with no avulsed fragment in contact with the bone bed, which is further divided into Type IIIA when avulsion involves only the ACL insertion and Type IIIB when avulsion withdraws entirely of the eminence. Zariczynj added Type IV to define a comminuted pattern of complete eminence avulsion fragment [7].

Generally, treatment of tibial eminence fracture is based on the type of fracture and associated knee injuries [5]. The literature supports conservative management for Type I by knee immobilization in near full extension [8]. If a closed reduction attempt by hemarthrosis aspiration and knee extension is not anatomical, surgical treatment for Type II is recommended [2], [5]. Closed reduction may also be attempted in Type III or IV, although the likelihood of success is low because the occurrence of soft tissue entrapment in this fracture pattern is high. Therefore, to avoid nonunion or malunion, surgical treatment of displaced tibial eminence fractures is required [9], [10].

In the past, displaced tibial eminence fractures were treated with open reduction and internal fixation. However, the arthroscopic approach has been more common recently [2], [11]. Despite that different arthroscopic methods have been developed [12], the ideal arthroscopic reduction and fixation technique for tibial eminence fractures is still uncertain [12], [13]. The purpose of this paper is to describe a modified technique for the reduction and fixation of tibial eminence avulsions by arthroscopic reduction and single tunnel pull-through suture fixation.

## 2 Case presentation

**Fig. 1:**
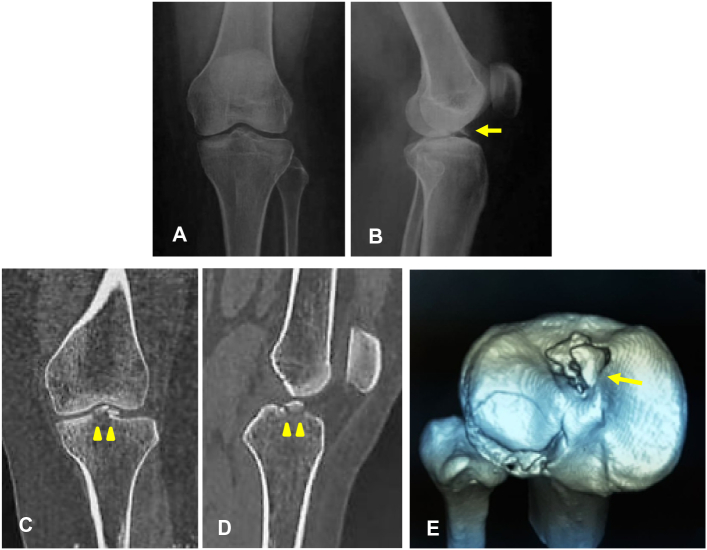
(A) The avulsion fracture of the ACL is not apparent on the frontal radiograph. (B) But lateral radiograph of the left knee confirms a displaced avulsion of the tibial eminence (yellow arrow). (C) Frontal section and, (D) Sagittal section of CT scan reveal displaced comminuted tibial eminence avulsion, (E) Three-dimensional CT image shows the avulsion fracture is comminuted involves entire of the ACL insertion (yellow arrow). (For interpretation of the references to colour in this figure legend, the reader is referred to the web version of this article.)

A 30-years-old female was admitted with a chief complaint of severe left knee pain after falling off a motorcycle and striking her left knee on the road. Her knee was swollen and painful right after the accident. She denied any history of previous knee deformity, injury, or surgery. Physical examination showed moderate left knee effusion with restricted knee joint range of motion from 0 to 90°. Anterior drawer test was positive without opening varus or valgus stress. An initial two-view X-ray radiograph of the left knee was obtained and denoted displaced tibial eminence avulsion. In preparation for surgery, a CT scan was performed to assess the displacement and comminution of avulsed fragment (Fig. 1).

### 2.1 Surgical technique

#### 2.1.1 Preparation

The patient underwent surgery 7 days after the accident at a major trauma referral hospital. The surgery was performed under epidural anesthesia. The position of the patient was supine with the knee in 90° flexion on a standard leg holder and thigh support. A pneumatic tourniquet was applied to reduce blood loss and improve visibility. High anterolateral and anteromedial portals were used. Initially, the arthroscopic cannula was inserted into the joint and the knee was then lavaged with thorough saline to wash the joint and remove the hematoma. Complete diagnostic arthroscopic then performed using the high anterolateral portal for assessment of meniscal tears, cartilage damage, loose bodies, and associated injuries. Clear intra-articular visualization was enhanced by adequate debridement.

Fracture debris and remaining blood clots were evacuated with a 3.5 mm shaver until the avulsed fragment and fracture site were exposed, and the ACL was probed to confirm its integrity. A 55°-adjusted ACL tibial guide then inserted through the high anteromedial portal and its tip laid on the distal part of the ACL so that the avulsed bone fragment was temporarily reduced (Fig. 2a).

**Fig. 2a:**
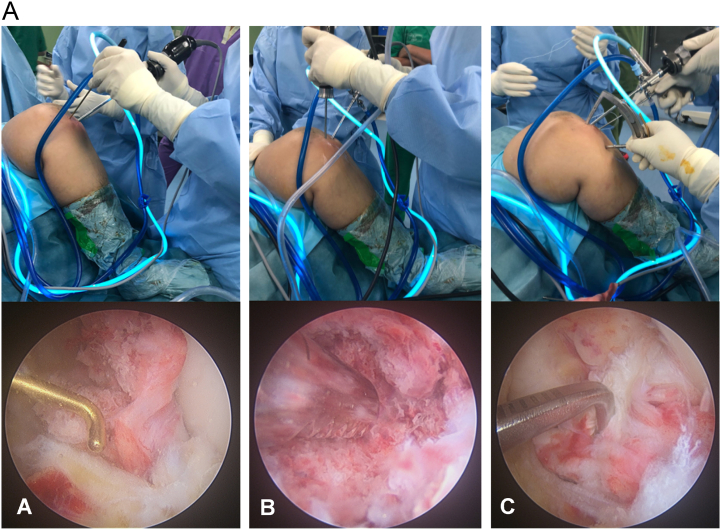
Left knee in 90° flexion viewed from the high anterolateral portal. (A) A probe is introduced from the high anteromedial portal to identify the ACL bundle, bone avulsion, and fracture bed. It is revealing comminuted fragment of bony avulsion. (B) A 3.5 mm shaver is used to remove fracture debris, loose fragments, and soft tissue interposed within the fracture bed. (C) An ACL tibial guide is then applied to the distal part of the ACL and temporarily reduces the avulsed fragment.

#### 2.1.2 Bundle sutures

A pair of non-absorbable ultra-high molecular weight polyethylene sutures (Hi-Fi Suture, Metric 5, Conmed, Linvatec) were used to stitch through the ACL bundle. First, employing the high anterolateral portal, a suture passer (Seahawk, Left, Conmed, Linvatec) was used to hook the ACL bundle just proximal to its tibial insertion on the fracture fragment, and the loop-stitch was passed outside the portal using an arthroscopic grasper. Then, another suture was passed through the high anteromedial portal using a suture passer or pre-bent cannula (Zone Specific, Right, Conmed, Linvatec) and pulled out through the high anteromedial portal. This maneuver was performed until the ACL bundle cross-tied perfectly. The double half hitches knot ligature was used on both sutures limbs and pushed to the base of the bundle knot using a knot pusher for ensuring a good hold of the ACL bundle (Fig. 2b).

**Fig. 2b:**
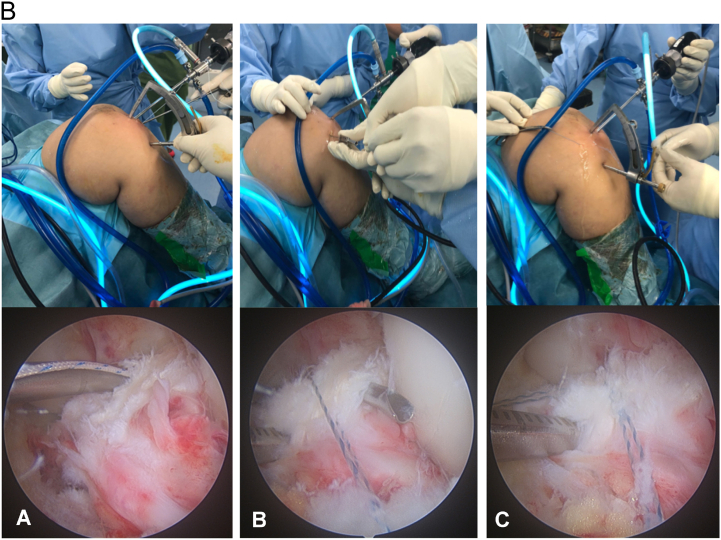
Left knee in 90° flexion viewed from the high anterolateral portal. (A) The ACL bundle is hooked by a suture passer, loaded with a non-absorbable suture to create a loop-stitch just proximal to its tibial insertion site on the fracture fragment. The loop then passed outside through the high anterolateral portal. (B) A pre-bent cannula is inserted through the high anteromedial portal to create another loop-stitch. (C) Both suture limbs are knotted double half hitches ligature, then the knot pushed to near the ACL bundle until the ACL bundle was firmly crossed-tied.

A 2-cm vertical incision was made over the anteromedial tibia at the level of the tibial tuberosity. Through the previous installed ACL tibial guide, a 1.25 mm pin wire was drilled aiming to the mid-coronal plane of the reduced avulsion bone fragment under direct arthroscopic visualization without going through the fracture fragment. Then, a single 4-mm tibial tunnel was drilled just below the fragment as close as possible. A non-absorbable No. 5 Ethibond suture loop was introduced through the tunnel, and its articular part was pulled outside the high anteromedial portal with an arthroscopic grasper. This loop was used to retrieve the previous suture limbs outside the anteromedial portal. A similar maneuver was performed while retrieving the suture limbs outside the high anterolateral portal. By withdrawing the No. 5 Ethibond loop through the tibial tunnel, the ends of all suture limbs were retrieved outside of the tunnel (Fig. 2c) (Fig. 3).

**Fig. 2c:**
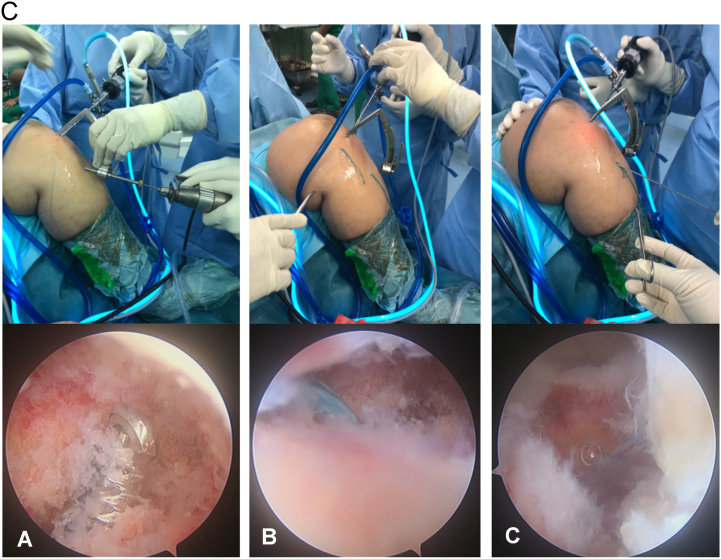
Left knee in 90° flexion viewed from the high anterolateral portal. (A) A tibial tunnel is created using a 4-mm drill, guided with an ACL tibial guide, and aimed just below the avulsion fragment. (B) An Ethibond suture loop is inserted through the tibial tunnel to retrieve the terminal suture limbs outside the high anteromedial portal. The same maneuver is conducted to the limbs outside the anterolateral portal (not shown). (C) The ends of all suture limbs are retrieved and delivered out of the tibial tunnel.

**Fig. 3:**
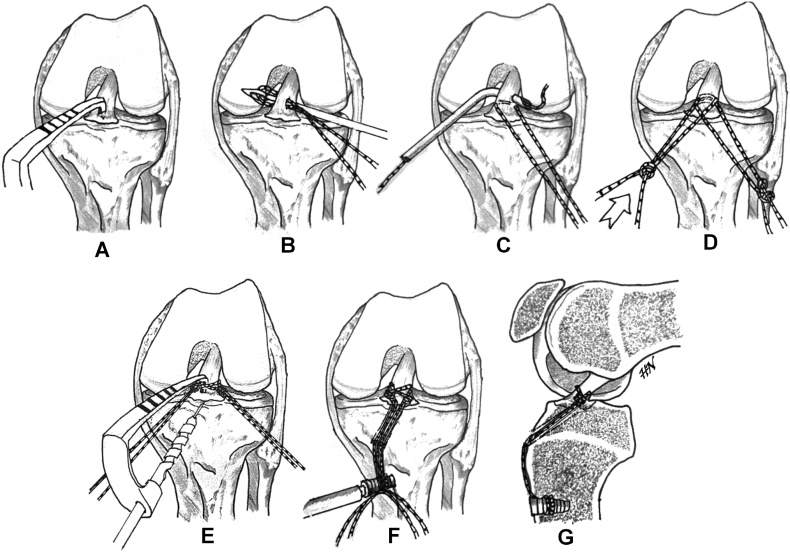
Schematic representation of the procedure. (A) A tip of ACL tibial guide is applied to the ACL bundle so that the fragment is temporarily reduced. (B) First loop suture is introduced to hook the ACL bundle with a suture passer. (C) Second loop suture is passed through a suture passer or pre-bent cannula. (D) The double half hitches knot ligature is used on both sutures until the ACL bundle is cross-tied firmly. (E) A single tibial tunnel is created using a 4-mm drill guided with the ACL tibial guide and aimed just below the avulsion fragment. (F) Through the tibial tunnel, both sutures are pulled down and tensioned and fixed over the anteromedial tibial cortex using a knotless suture anchoring screw. (G) Tibial eminence avulsion is well reduced and fixed.

#### 2.1.3 Reduction and fixation

**Fig. 4:**
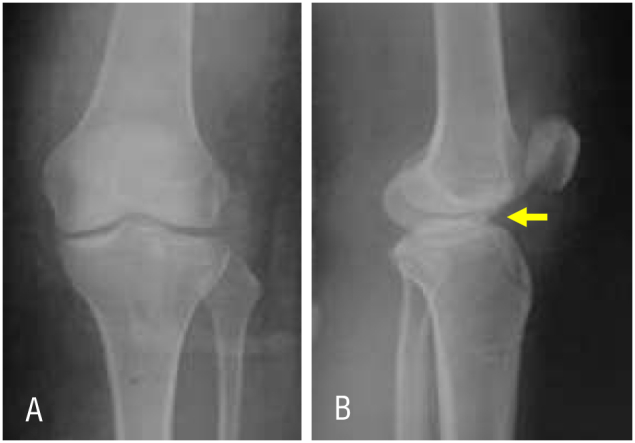
(A and B). Postoperative radiologic X-ray of the left knee shows anatomical reduction of the fragment (yellow arrow). (For interpretation of the references to colour in this figure legend, the reader is referred to the web version of this article.)

The anatomic bone avulsion reduction was achieved and evaluated arthroscopically by pulling down the sutures limbs. With firm tensioning, the 4 suture limbs were then fixed distally over the anteromedial tibial cortex using a knotless suture anchoring screw (PopLock 4.5 mm, Conmed, Linvatec). Knee stability was evaluated with an anterior drawer test. The tension of the ACL bundle after fixation was confirmed arthroscopically with a probe under direct visualization. The postoperative radiologic X-ray showed well-reduced bone avulsion fragment (Fig. 4).

#### 2.1.4 Post-operative management and follow up

**Fig. 5:**
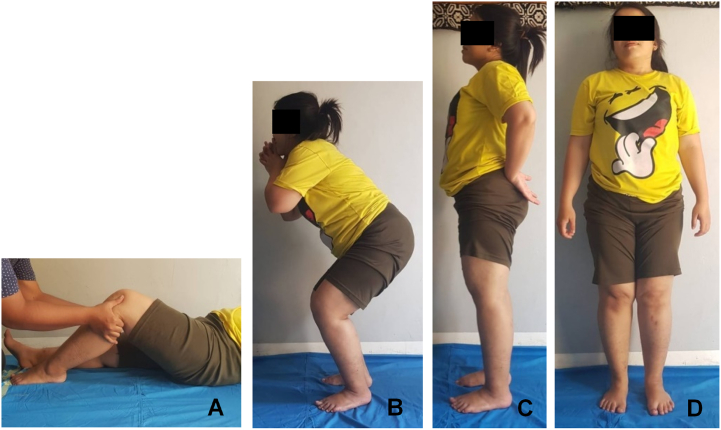
At 6 months follow up. (A) Stable knee tested with anterior drawer test, (B) stable knee at squatting, (C) at full standing, (D) good wound healing and no knee pain complained.

The knee joint was immobilized in full extension within a knee brace for 2 weeks after surgery. After 2 weeks, partial weight bearing supported by a crutch was allowed as tolerated with gradual range-of-motion exercise. Full weight bearing was permitted at 6 weeks postoperatively. To avoid muscle atrophy, isometric quadriceps muscle exercise was performed through the immobilization period. At 6 months follow-up, the patient reported no complaint of knee pain or instability (Fig. 5). The study then reported in line with the SCARE Criteria guidelines [14].

## 3 Discussion

The avulsion of ACL is comminuted in about a quarter of all cases [15], and operative treatment is highly recommended for this type IV avulsion due to the high occurrence of soft tissue entrapment in this fracture pattern, and associated injuries [4], [9]. Displaced tibial eminence fracture may develop nonunion, limitation of range motion, and instability [1]. This injury is more complicated in adult when compared with pediatric patients [3]. In comparison with children and adolescents, the overall treatment results are less desirable in adults [16]. But despite the patient's age, anatomic reduction and stable fixation are required to promote fracture healing and restore normal biomechanics of the knee joint [15].

Conventionally, open surgery was performed for displaced tibial eminence avulsion fractures, but due to several drawbacks and complications, such as more soft tissue damage, higher postoperative pain, longer hospital stay, and delayed rehabilitation [8], arthroscopic reduction and internal fixation became the treatment of choice [2]. Furthermore, it enables direct viewing of intra-articular injuries, easier diagnosis, precise fracture fragment reduction, treatment of associated soft-tissue injuries, and removal of loose bodies [8].

Many various arthroscopic-assisted reduction and internal fixation procedures have been documented for tibial eminence fractures [17], although screw fixation and suture fixation are the most commonly described, and both have provided good results [13]. Screw fixation on the avulsed fragment has been proved to have high strength in fracture stabilization [18], but when the fracture fragment is comminuted or small in size, screw fixation is technically impossible [10]. Coupled with the risk of screw impingement which results in loss of knee extension and the requirement for screw removal [18], [19], arthroscopic reduction and suture fixation has become the preferred approach for displaced or comminuted tibial eminence avulsion fractures [17]. Bong et al [20] conducted a biomechanical investigation using cadaveric knee specimens to assess the strength of sutures against cannulated screw fixation under a constant load and found that suture fixation was stronger than screw fixation.

With many reports of excellent outcomes, the arthroscopic pull-out suture technique appears to have gained prominence over another suture technique of fixation. Without the necessity for provisional fixation of the avulsed fragment, our technique uses modified 2-point suture fixation through a single tibial tunnel with a suture levering technique. The benefits of our method are the elimination of multiple drilling of tibial tunnel, avoiding penetration drilling that can split the fragment, and stable tensioning with knotless anchor distal screw fixation. We noted some limitations of our technique. With this procedure, the suture tension threshold could not be ascertained and the technique is technically demanding. It might also be noted that our method has only been performed in the clinical setting. Further biomechanical studies are needed to confirm the most optimal fixation method used (Table 1).

**Table 1:** Pearls, advantages, and limitations of technique.

Pearls
High anterolateral and anteromedial portals give better viewing and access for suture passage
The suture movement and tension of the ACL bundle can be directly visualized arthroscopically


Advantages
Does not require provisional fixation with K-wire
It no necessary for multiple drilling to create the tibial tunnel


Limitations
The suture tension threshold for this procedure could not be ascertained
Technically challenging knee arthroscopy procedure

## 4 Conclusion

Despite some limitations, this technique simplifies anatomic reduction and fixation with single tunnel placement. Moreover, we conclude that this proposed modification technique is a viable alternative that requires less operative time.

## Funding

None declared.

## Ethical approval

This study has been approved by Hospital Ethical Committee.

## Consent

Written informed consent was obtained from the patient for publication of this case report and accompanying images. A copy of the written consent is available for review by the Editor-in-Chief of this journal on request.

## CRediT authorship contribution statement

HN: perform surgery, perform the literature review and data collection, designing and writing the manuscript, revision and approval of final manuscript.

KDH: perform surgery, concept and design the study, contributed to the manuscript writing, revision and approval of final manuscript.

## Research registration

Not applicable.

## Guarantor

Kukuh Dwiputra Hernugrahanto.

## Provenance and peer review

Not commissioned, externally peer-reviewed.

## Declaration of competing interest

None declared. The authors have no financial, consultative, institutional, and other relationship that might lead to bias or conflict of interest.
